# Accuracy of seizure semiology obtained from first-time seizure witnesses

**DOI:** 10.1186/s12883-018-1137-x

**Published:** 2018-09-01

**Authors:** Taim A. Muayqil, Mohammed H. Alanazy, Hassan M. Almalak, Hussain Khaled Alsalman, Faroq Walid Abdulfattah, Abdullah Ibrahim Aldraihem, Fawaz Al-hussain, Bandar N. Aljafen

**Affiliations:** 1 Division of Neurology, College of Medicine, King Saud University, PO Box 7805 (38), Riyadh, 11472 Saudi Arabia; 2 College of Medicine, King Saud University, PO Box 7805 (38), Riyadh, 11472 Saudi Arabia

**Keywords:** Seizure semiology, History, Seizure witness

## Abstract

**Background:**

Little is known of how accurately a first-time seizure witness can provide reliable details of a semiology. Our goal was to determine how accurately first-time seizure witnesses could identify key elements of an epileptic event that would aid the clinician in diagnosing a seizure.

**Methods:**

A total of 172 participants over 17 years of age, with a mean (sd) of 33.12 (13.2) years and 49.4% female, composed of two groups of community dwelling volunteers, were shown two different seizure videos; one with a focal seizure that generalized (GSV), and the other with a partial seizure that did not generalize (PSV). Participants were first asked about what they thought was the event that had occurred. They then went through a history-taking scenario by an assessor using a battery of pre-determined questions about involvement of major regions: the head, eyes, mouth, upper limbs, lower limbs, or change in consciousness. Further details were then sought about direction of movement in the eyes, upper and lower limbs, the side of limb movements and the type of movements in the upper and lower limbs. Analysis was with descriptive statistics and logistic regression.

**Results:**

One hundred twenty-two (71.4%) identified the events as seizure or epilepsy. The accuracy of identifying major areas of involvement ranged from 60 to 89.5%. Horizontal head movements were significantly more recognized in the PSV, while involvement of the eyes, lateralization of arm movement, type of left arm movement, leg involvement, and lateralization of leg movement were significantly more recognized in the GSV. Those shown the GSV were more likely to recognize the event as "seizure" or "epilepsy" than those shown the PSV; 78 (84.8%) vs 44 (55.7%), (OR 0.22, *p* < 0.0001). Younger age was also associated with correct recognition (OR 0.96, P 0.049). False positive responses ranged from 2.5 to 32.5%.

**Conclusion:**

First-time witnesses can identify important elements more than by chance alone, and are more likely to associate generalized semiologies with seizures or epilepsy than partial semiologies. However, clinicians still need to navigate the witness’s account carefully for additional information since routine questioning could result in a misleading false positive answer.

## Backgound

The occurrence of a seizure is a relatively common event that can develop in about 10% of the population [1]. The diagnosis of a seizure or epilepsy relies heavily on the description of the event by a witness. The seizure description of the clinically observed behavioral motor, sensory or psychiatric signs that occur in a pattern or sequence is known as the semiology, understanding the semiology is useful in diagnosis and localization [2–4]. The task of reporting the details of a semiology usually falls on the shoulders of a bewildered bystander, and when it involves a first-time seizure victim, the witness is likely a first-timer as well. Obtaining an appropriate account of events from a witness is a crucial component of the patient assessment, particularly since most seizures do not last long enough to be identified by first responders, and patients are also unable to provide helpful information because they present to the emergency in a post-ictal and confused state with no memory of the event [1]. Although assessment tools like routine electroencephalogram or brain imaging can help, they are not always diagnostic [1, 5]. Also, while inquiring about seizure risk factors or looking for clinical signs that suggest a seizure has occurred are important [1, 4], knowing the semiology details provides additional localizing and diagnostic value that can aid in management. For example, recognizing a primary generalized semiology in a young female would raise the suspicion for juvenile myoclonic epilepsy, which is known to respond to certain drugs and worsen with others [1].

The physician carries the responsibility of taking the appropriate history in order to extract useful information. Non-specialists and trainees may be more concrete in their history taking technique, which creates a challenge in obtaining diagnostic information given the wide variability in how witnesses report their experiences. An accurate interpretation of the history is the most important step in patient evaluation [6], and it takes years of experience for a physician to acquire the skills and knowledge to differentiate relevant information from not. Therefore, our goal with this study was to assess the accuracy of the accounts of first time witnesses of seizures when history is obtained through a routine battery of questions that probe common seizure semiology components. First, we identify if a first time seizure witness could recognize the occurrence of an epileptic event; second, we examine what seizure semiology components are recognized and if there will be a difference in responses according to seizure type, after viewing two different seizure semiology videos, one generalized and the other partial.

## Methods

### Participants

In this experiment, we recruited willing volunteers from the community over 17 years of age by convenience sampling. Recruitment was done between June and August 2017, in Riyadh, Saudi Arabia. Those with a personal or family history of epilepsy or workers of any kind in the healthcare field were excluded from the study. To screen for this, and preserve participant unawareness of what is about to be watched, our question about a personal or family history of epilepsy was embedded within a long list of other neurological conditions. Information was also gathered regarding age, education level, marital status, and place of residence.

### Materials

Two different seizure videos obtained from the reference “Manual of Neurological Signs” [7] were used to assess participant responses. Two neurologists and an epileptologist chose the videos based on the clarity of the features to be assessed in the study. The first video showed a generalized tonic-clonic convulsion that had a focal onset. The patient was awake in her bed initially, and then developed a brief stare. This was followed by head deviation upward and to the right, with widely opened eyes that deviated in the same direction. The mouth was open with incoherent vocalization; this was then followed by tonic posturing of upper and lower limbs bilaterally and a subsequent clonic phase. The patient was not awake at the end of the video and did not interact with anyone surrounding her. The whole event lasted 65 s. For brevity, this video was referred to as the generalized seizure video (GSV). The second video showed a different patient who, while asleep, began to develop left sided mouth clonic twitching, followed by head clonic movements to the left and leftward eye deviation. This was then followed by the development of clonic movements of the left arm and leg. The patient did not exhibit any ability to communicate or interact with her surrounding and was not awake at the end. The entire event lasted 45 s. This second video was referred to as the partial seizure video (PSV).

### Procedure

Participants were pseudo-randomized at the time of recruitment to balance gender distribution into two groups. Each group was shown one of the two videos and all were asked the same battery of questions. These questions were designed in Arabic by three neurologists (TAM, MHA, and BNJ) and pilot tested on seven individuals matching the target population to check the comprehensibility of the questionnaire and eliminate ambiguous questions. The assessments occurred within the confines of the academic institute and videos were viewed only once per participant on a laptop monitor.

The interviewer first asked an open question at the end of each video on what the viewer thought the event that happened to the patient was. Those that referred to it as epilepsy or seizure were considered correct. Participants were then asked a series of questions about the semiology, these questions were designed without reference to the two videos and not modified according to seizure type. This ensured that both groups were exposed to the same battery of questions in order to mimic all the inquiring that occurs during an actual history-taking scenario. Identification of a feature that was independently identified by all three neurologists or present in the reference’s seizure description was considered a correct response. Patients were asked “yes/no” questions on whether there was involvement of the head, eyes, mouth, upper limbs, lower limbs, consciousness, or the presence of vocalization. During this phase of questioning, answers were considered correct if they answered “yes” to any of the above regions that had exhibited epileptic involvement of any kind without going into the specifics of the involvement such as direction or type of abnormal movement. For consciousness, the answer was correct if it was “yes” to “*Was there loss of consciousness?*” The answer to the question about vocalization was correct if it was identified as “present” in the GSV and identified as “absent” in the PSV. Participants were also asked if they noticed a focal region where the seizure started (which happened in both videos) or not.

Participants were than asked about details of movements: direction of movement in the main body parts queried (head, eyes, upper and lower limbs), the lateralization of limb movements (right, left or both sides), and the type of movement in the upper and lower limbs (tonic, clonic or both). With regard to questions on the latter, we considered a correct response in the GSV when a participant indicated either tonic or clonic activity. At the end, we inquired about an estimation of how long the event lasted. All participants had to sign consent before starting the interview, and an ethical approval was obtained from the internal review board at the college of medicine at King Saud University.

### Analysis

Descriptive statistics were used for assessing demographic features and percentage of individuals with correct responses to any of the queried features. Univariate and multivariate logistic regression analysis was done; identification of the event as a seizure was the dependent variable and the demographic variables of age, sex, marital status and type of seizure shown represented the independent variables. The significance of adding the video type viewed by participants to a model containing only the demographic variables was also tested. Proportions of false positive responses to queries about semiology details were also tabulated. STATA statistical software was used.

## Results

Table 1 shows the demographic characteristics and the proportions of correct responses to whether the event was a seizure, and to which body part exhibited involvement for each of the six main shared semiology features in both videos. Only two individuals resided in a rural area. Three individuals requested the videos be stopped before completion at 34 and 36 s for the PSV, and at 75 s for the GSV; their responses were included in the analysis, as the full semiology had developed by the time a stop was requested. Regarding the responses to each video, there was a slight difference in age, with the PSV group being older by about 6 years. The differences in gender, marital status and mean years of education were not significant (Table 2).

**Table 1:** Demographic distribution and identification of shared seizure features between the two videos

	*N* = 172
Female	85 (49.42%)
Age, years (SD)	33.12 (13.2)
Years of Education, years (SD)	13.3 (3.9)
Married, yes	93 (54.7%)
Number and percentage of subjects who correctly identified the following features:	
• The event was a seizure	122 (71.4%)
• Involvement of head/neck^a^	154 (89.5%)
• Involvement of eyes^a^	108 (63.2%)
• Involvement of mouth^a^	143 (83.1%)
• Involvement of upper limb(s)^a^	142 (83%)
• Involvement of lower limb(s)^a^	118 (68.6%)
• There was loss of consciousness^a^	102 (60%)
• Presence or absence of vocalization^b^	87 (51.2%)
• Starting region of the seizure	143 (86.6%)

^a^Represents a shared feature between both videos. ^b^ Considered correct if identified vocalization in GSV and identified no vocalization in PSV

**Table 2:** Correct responses to each video

	GSV *N* = 92	PSV *N* = 80	*p* Value
Female	42 (45.7%)	43 (53.8%)	0.36
Age	30.1 (10.9)	36.6 (14.6)	0.002
Years of Education	12.9 (3.7)	13.6 (4.2)	0.25
Married	46 (50.5)	47 (59.5)	0.28
Correctly identified:			
• The event as a seizure	78 (84.78%)	44 (55.7%)	< 0.0001
• Involvement of head	79 (89.9%)	75 (93.8%)	0.13
• Vertical head movement	17 (18.5%)	–	–
• Horizontal head movement	29 (31.5%)	55 (68.8%)	< 0.0001
• Involvement of eyes	68 (73.9%)	40 (50%)	0.002
• Direction of eye movement	64 (69.6%)	36 (45%)	0.002
• Involvement of the mouth	75 (81.5%)	68 (85%)	0.68
• Involvement in an arm (s)	78 (84.8%)	64 (80%)	0.43
• Lateralization of arm movement	72 (78.3%)	35 (43.8%)	< 0.0001
• Type of movement (right arm)	76 (82.6%)	–	–
• Type of movement (left arm)	71 (77.2%)	42 (52.5%)	0.001
• Involvement in a leg (s)	76 (82.6%)	42 (52%)	< 0.0001
• Lateralization of leg movement	72 (78.3%)	20 (25%)	< 0.0001
• Type of movements (right leg)	72 (78.3%)	–	–
• Type of movements (left leg)	60 (65.2%)	23 (28.8%)	0.06
• Loss of consciousness	60 (65.9%)	42(53.2%)	0.06
• Vocalizations	27 (29.7%)	–	
• A focal start for the event	72 (78.3%)	71 (88.8%)	0.16
• Duration of seizure	Mean (SD) 97.4 (97.5), Median 60 (Seconds)	Mean (SD) 52.9 (49.1), Median 37.5 (Seconds)	

Table 2 demonstrates the differences in correct responses to the general questions and to specific involvement of different body regions and types of movements. Generally, the correct identification of semiology components was less in the PSV group, with the exception of head movement along the horizontal axis being significantly more identified in the PSV. In the GSV, participants were more likely to identify involvement of the eyes, direction of eye movement, lateralization of arm movement, type of movement in the left arm, involvement in a leg, and lateralization of leg movement. Vocalizations were difficult to detect by observers in the GSV 27 (29.7%). More participants identified tonic than clonic movements. In the right upper limb, tonic and clonic movements were identified by 44 (47.8%) and 32 (34.8%), respectively. In the left upper limb, tonic and clonic movements were identified by 44 (47.8%) and 27 (29.4%), respectively. In the right lower limb, tonic and clinic movements were identified by 40 (43.5%) and 32 (34.8%), respectively. In the left lower limb, tonic and clonic movements were identified by 35 (38%) and 25 (27.2%), respectively.

Logistic regression analysis is demonstrated in Table 3. Participants were significantly more likely to identify the event as a seizure or epilepsy depending on the type of seizure viewed, with the GSV more likely to be called a seizure or epileptic event (*p* < 0.0001). Younger individuals were also more likely to identify the event correctly, and this finding just barely reached statistical significance. A hierarchal approach to the logistic regression showed that adding the type of seizure shown improved the prediction of the model containing the demographic variables with likelihood ratio (χ^**2**^) 15.4 and *p* value of 0.0001.

**Table 3:** Logistic regression. The odds of describing the event as a seizure or epilepsy in relation to demographic variables and the seizure video shown

	Univariate		Multivariate: LR (χ^2^) 26.4, *p* = 0.0001	
	OR (95% CI)	*p* value	OR (95% CI)	*p* value
Age	0.97 (0.941–0.992)	0.01*	0.96 (0.93–0.9998)	0.049*
Years of education	1.02 (0.938–1.109)	0.65	0.99 (0.896–1.093)	0.83
Male gender	1.25 (0.642–2.423)	0.514	1.21 (0.568–2.597)	0.62
Married	0.79 (0.402–1.545)	0.49	1.95 (0.699–5.433)	0.2
PS Video	0.23 (0.11–0.464)	< 0.0001*	0.22 (0.098–0.481)	< 0.0001*

* Statistically significant result

Finally, we looked at the responses of participants who viewed the PSV to queries about events that did not occur (Table 4). The most frequent false positive response was of seizure involvement on the contralateral upper and lower limbs, followed by discriminating tonic from clonic activity.

**Table 4:** False positive answers for the different movements which did not occur in the PSV

Movement	*N* = 80
Head movement along vertical axis	7 (8.5%)
Vocalization	2 (2.5%)
Tonic/clonic movements in the right upper limb	26 (32.5%)
Tonic/clonic movements in the right lower limb	18 (22.5%)
Tonic activity left upper limb	15 (18.8%)
Tonic activity left lower limb	14 (17.5)

## Discussion

Little attention has been given to the accuracy of reports from first time seizure witnesses thus far. In this study, we have demonstrated that seizure witnesses are able to identify key elements that aid the clinician in considering a seizure diagnosis in the majority of encounters. These witnesses are also likely to call these events “seizures” or “epilepsy”. While we found that first-time witnesses can also recognize the involvement of different body regions, the exact detail of this involvement appears to be more difficult to accurately report. Participants were clearly less assertive about calling the event a seizure when they were shown the PSV, and their detection of its semiology components was also less frequent. These findings suggest that in the mind of the average individual, seizures are a generalized event. We also found that there was a high identification rate of limb and head movements in general, this is corroborated by similar findings obtained in a study that researched experienced seizure witnesses which included relatives, friends or care-givers [8].

Interestingly, the direction of eye movement was more frequently identified in the GSV than the PSV. This is probably because the GSV patient was lying more upright, and the eyes moved both upward and to the side, any of which were considered a correct response, thus increasing the probability of obtaining a correct answer. In the PSV, the patient was on her back during the whole event and had prominent left head deviation and left facial twitching that may have distracted the viewer from seeing the eyes. This is also supported by the fact that, in the same video, eye involvement was recognized less frequently than head and mouth involvement (Table 2.). Similarly, only a third of our first-time seizure witnesses were able to identify the vocalization in the GSV. Although correct identification of vocalization and direction of eye movements have been previously described [3], this too was a finding in experienced witnesses. The videos in our study did not focus on the eyes enough to allow accurate reporting from a novice. We intentionally kept the questioning restricted to gross movements to look for the essential descriptions that would enable physicians to recognize that a seizure had occurred, and because observations of more subtle features like automatisms, lip movements or staring episodes are more difficult to identify [8, 9]. Previous evaluations of experienced seizure witnesses have also showed that different semiologies can be associated with different levels of reporting accuracy [3].

While level of education was previously found to be an important factor in providing semiology details in experienced witnesses [8], it was not significant in our study of first-time witnesses. Younger age being associated with higher odds of calling an event a seizure is probably the result of increasing media and societal awareness that has lead to higher health literacy in younger generations, consistent with the fact that medical knowledge is associated with more accurate seizure descriptions [10]. The influence of gender was not significant in our study, which is consistent with a previous study that compared descriptions of syncopal with epileptic events and found no influence from gender [11]. Nonetheless, the majority of witnesses in our study recognized involvement of multiple body regions, and their observations were more likely to be correct in a generalized seizure.

There appears to be a small tendency by some participants to falsely describe movements that could lead an assessor to believe it was a generalized event. In fact, false positive responses occurred previously among nine out of 20 participants in a study that looked at the seizure descriptions from volunteers with varying medical backgrounds, [12]. Another study that looked at the accounts of experienced witnesses suggests that inaccuracies are more likely to occur in reporting convulsive than non-convulsive events [3] and, similar to our study, it was highest when addressing limb movements. It seems that while witnesses will recognize limb movements frequently, there is a small trade-off that a small proportion will report inaccurate information. While experienced observers have been found to recall the presence or absence of certain semiology components, they too have mistaken the side of involvement or even believed the involvement to be bilateral [8].

Insistent questioning or restricting the witness to provide a “yes” or “no” response could result in misleading information or even in increasing confidence in the false answer if perpetuated [13–15]. However, witnesses usually do not spontaneously offer all the required information [12], and obtaining a useful account relies on the clinician’s skills; going through a battery of routine questions may not be applicable in all situations [16].

In this study, participants described the findings in two videos in a controlled and reassuring environment where no safety concerns are required for the victim. A real-world seizure however is a very dramatic event to a first-time witness; the emotional impact will affect observation details. While accuracy and consistency for recall of witnessed events is addressed frequently in events of a legal nature, witnessed medical conditions and the impact it has on clinical history taking has not been similarly studied. Recollection of emotional events and their sequence has been found to be variable, incomplete, and dependent on the personal consequences of the witness [17, 18]. This is also an important consideration when assessing the perception of lapsed time, while the mean estimated time reported here was higher than the actual, it is probably less than the estimated time provided by witnesses of an actual event.

One advantage to our study design is that we used a battery of standard questions usually used during witness interviews to mimic the actual history taking process, where the physician has to ask questions about events that both did and did not occur, especially since witnesses do not spontaneously provide all the required information without prompting [12]. Some limitations include that participants had to determine loss of consciousness from watching the videos; the segments did not contain any part that specifically assessed consciousness. The assessment of eye direction might have been difficult to determine because they were not the main focus of the camera angles. Reevaluating the ability of the participants to recount the semiologies after a time period from 30 min to 1 hour might offer a more precise mimicry of recounting in clinical settings [11]. The responses we obtained were not confounded by emotional stressors, it is yet to be seen if real world accuracy would be higher or lower than that found here. If anything, this information provides us with the reporting potential of first time witnesses. Since the goal of the study was to focus on what semiology elements would be recalled after witnessing a seizure, further research investigating the reports of first time witnesses to non-epileptic events or more difficult semiology types such as automatisms, could supplement the findings in this study.

## Conclusion

In conclusion, the ability to identify major seizure semiology elements by inexperienced witnesses is more than chance alone, based on these results, there is a good chance of obtaining an informative description from first time witnesses that would help diagnose a seizure or even aid in lateralization. However, false positive information may be inadvertently given, especially when inquiring about limb movements, and the clinician still needs to use his or her history taking skills in retrieving the additional information that would support the diagnosis or aid with lateralization.

## Abbreviations

GSV: Generalized Seizure Video
PSV: Partial Seizure Video
